# Inhibition of HIV-1 integrase nuclear import and replication by a peptide bearing integrase putative nuclear localization signal

**DOI:** 10.1186/1742-4690-6-112

**Published:** 2009-12-05

**Authors:** Aviad Levin, Ayelet Armon-Omer, Joseph Rosenbluh, Naomi Melamed-Book, Adolf Graessmann, Elisabeth Waigmann, Abraham Loyter

**Affiliations:** 1 Department of Biological Chemistry, The Alexander Silberman Institute of Life Sciences, The Hebrew University of Jerusalem, Jerusalem 91904, Israel; 2 Institut fur Molekularbiologie und Biochemie, Free University of Berlin, Germany; 3 Max F. Perutz Laboratories, University Departments at the Vienna Biocenter, Institute of Medical Biochemistry, Medical University of Vienna, Austria; 4 Ziv Medical Center, Zefat 13100, Israel

## Abstract

**Background:**

The integrase (IN) of human immunodeficiency virus type 1 (HIV-1) has been implicated in different steps during viral replication, including nuclear import of the viral pre-integration complex. The exact mechanisms underlying the nuclear import of IN and especially the question of whether it bears a functional nuclear localization signal (NLS) remain controversial.

**Results:**

Here, we studied the nuclear import pathway of IN by using multiple *in vivo *and *in vitro *systems. Nuclear import was not observed in an importin α temperature-sensitive yeast mutant, indicating an importin α-mediated process. Direct interaction between the full-length IN and importin α was demonstrated *in vivo *using bimolecular fluorescence complementation assay (BiFC). Nuclear import studies in yeast cells, with permeabilized mammalian cells, or microinjected cultured mammalian cells strongly suggest that the IN bears a NLS domain located between residues 161 and 173. A peptide bearing this sequence -NLS-IN peptide- inhibited nuclear accumulation of IN in transfected cell-cycle arrested cells. Integration of viral cDNA as well as HIV-1 replication in viral cell-cycle arrested infected cells were blocked by the NLS-IN peptide.

**Conclusion:**

Our present findings support the view that nuclear import of IN occurs via the importin α pathway and is promoted by a specific NLS domain. This import could be blocked by NLS-IN peptide, resulting in inhibition of viral infection, confirming the view that nuclear import of the viral pre-integration complex is mediated by viral IN.

## Background

Active nuclear import begins in the cytoplasm with recognition of the transported cargo molecules by nuclear transport receptors designated as importins [1]. Proteins targeted to the nucleus contain a specific amino acid sequence, termed nuclear localization signal (NLS), which is recognized by either a member of the importin α family, or directly by importin β. The resultant complex then interacts with the nuclear pore complexes (NPCs), through which it is subsequently transported into the nucleus [2]. This nuclear translocation machinery is highly conserved among lower and higher eukaryotes [3].

Human immunodeficiency virus type 1 (HIV-1) belongs to the lentivirus family, which in contrast to other retroviruses can infect terminally differentiated cells [4,5]. The capability of HIV-1 to infect cell-cycle arrested cells has been ascribed to the ability of its pre-integration complex (PIC) [6,7] to translocate across the nuclear envelope via the NPC [1]. The karyophilic properties of the viral PIC have been attributed mainly to three viral proteins: matrix (MA), Vpr, and integrase (IN) [8-10]. The cellular Lens Epithelium-Derived Growth Factor p75 (LEDGF/p75) protein as well as the DNA flap structure of the viral cDNA have also been implicated in promoting the translocation of the PIC into nuclei of virally infected cells [11-13]. Yamashipa et al. have proposed that the HIV capsid protein plays a crucial role in controlling the nuclear import of the HIV genome [14]. However, despite these extensive studies and numerous reports, the nuclear import mechanism of the PIC and the involvement of viral or cellular factors driving such a process remain unclear and controversial [15].

The HIV-1 IN protein consists of 288 amino acids and three functional domains: the N-terminal domain (residues 1-50), which bears a zinc-binding motif [16,17]; the central core domain (residues 51-212), which includes the catalytic DDE motif [18-20]; and the C-terminal domain (residues 213-288), which has been shown to non-specifically bind the DNA [19-21]. To achieve integration of the viral DNA into the host chromosome, the IN must be translocated into the nuclei of infected cells [15].

Various studies have showed that IN is a karyophilic protein. Transfection of cultured mammalian cells with expression vectors bearing IN results in nuclear accumulation of the encoded protein [22]. Import of fluorescently labeled IN into the nuclei of digitonin-permeabilized mammalian cells was shown to be ATP- and temperature-dependent; and this import could be blocked by the addition of unlabeled IN, clearly indicating an active, receptor-mediated process [23,24]. Based on the ability of recombinant IN protein to bind *in vitro *to importin α and the ability of a peptide bearing the prototypic simian virus 40 T-antigen NLS (SV40-NLS) to block such binding, as well as nuclear import, nuclear transport of IN has been suggested to occur via the importin α pathway [8,23]. Moreover, interaction of IN with the importin α family has recently been reported [25].

The possibility of the IN protein being carried into the cell's nuclei by other cellular components has also been suggested [13,26,27]. The LEDGF/p75 was initially implicated in mediating the nuclear import of IN [13]. However, studies on the specific contributions of LEDGF/p75 demonstrated that it facilitates the interaction between IN and nuclear chromatin, but is not directly involved in the import process [28]. An interaction with importin 7, via a sequence located at the C terminus of IN [26], has been proposed. However conflicting results have been obtained regarding the necessity of this receptor [29,30]. Furthermore anti-importin 7 antibodies did not block nuclear import of IN [25]. More recently, the involvement of the transportin-SR2 (TNPO3) in the nuclear import of IN has been suggested [27]. This conclusion is based mainly on experiments showing that the knockdown of transportin-SR2 (TNPO3) resulted in the reduction of nuclear cDNA [27].

In the present work, we further confirm and emphasize the role that importin α plays in promoting nuclear import of the viral IN and thus in virus infection. Multiple approaches and various experimental systems such as transfected mammalian and yeast cells as well as virally infected cells have been used to answer the question of whether nuclear import of IN may be mediated by its own NLS via interaction with importin α. Our results clearly demonstrate that IN accumulates within wild-type yeast cell nuclei, but fails to do so in importin α-defective yeast mutants (*srp1-31*) [31]. A full-length IN, as well as a peptide bearing the IN amino acid sequence 161-173 (NLS-IN), interacted *in vivo *with mammalian importin α, as demonstrated by a bimolecular fluorescence complementation (BiFC) assay [32] in yeast. The involvement of amino acids 161-173 in mediating nuclear import of IN was also demonstrated by microinjection and transfection experiments in cultured mammalian cells. Furthermore, the putative NLS-IN peptide inhibited nuclear accumulation of IN as well as of cDNA in IN-transfected and virally infected cells. This appears to be due to the ability of the NLS-IN peptide to compete for the interaction between the viral IN and the cellular importin α. This peptide has also been found to significantly inhibit HIV-1 replication in TZM-bl cells and inhibit the integration of viral cDNA in infected cells. Thus, the present results support our [23] and others' previous results [33] claiming that the IN protein contains a specific functional NLS sequence, which is located between amino acids 161 and 173 and which confers to this protein the karyophilic property required to ensure productive viral infection.

## Results

### The NLS-IN peptide is functional in transfected and microinjected mammalian cells as well as in yeast cells

**Figure 1:**
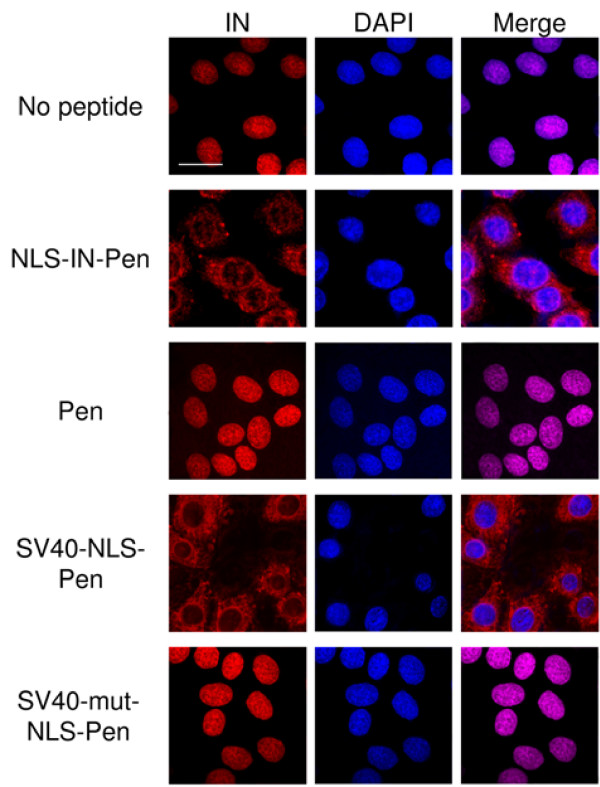
**Immunostaining experiments for intracellular localization of IN in transfected cells**. HeLaP4/IN-expressing cells were generated by stable transfection into HeLaP4 cells using pcDNA3.1 plasmid bearing the full wt IN gene. Cells were fixed and immunostained using 1:100 rabbit a-IN and second antibody, Cy3-conjugated anti-rabbit antibody as described in Methods. Staining of IN (red) and DAPI (blue) was observed under confocal microscope. Bar 10 μm.

The results in Fig. 1 clearly show that in stably transfected aphidicolin treated cell-cycle arrested HeLa/P4 cells, HIV-1 IN accumulates within the nuclei, confirming previously published results [22]. We next evaluated the ability of the NLS-IN peptide [23] to block the nuclear import of IN. However, the NLS-IN peptide was found to be cell-impermeable (not shown). Addition of the cell-permeable penetrating peptide (Pen-peptide) [34] sequence to the NLS-IN converted the latter to a cell-permeable peptide (not shown) which was designated NLS-IN-Pen. No toxic effect was exerted by this peptide during the time of the experiment, as estimated by MTT assay (data not shown), thus allowing for studies on its effect in cultured cells. As can be seen (Fig. 1), following the incubation of the transfected cells with the NLS-IN-Pen peptide, very little - if any - IN was intranuclear; most of it was located within the cytoplasm, clearly demonstrating the inhibition of nuclear import. Incubation with the Pen-peptide alone did not have any effect on nuclear import of IN (Fig. 1), strongly indicating a specific effect of the NLS-IN. The same results were obtained when the transfected cells were incubated with a cell-permeable SV40-NLS-Pen peptide (Fig. 1), indicating an importin α-dependent nuclear import pathway [35].

**Figure 2:**
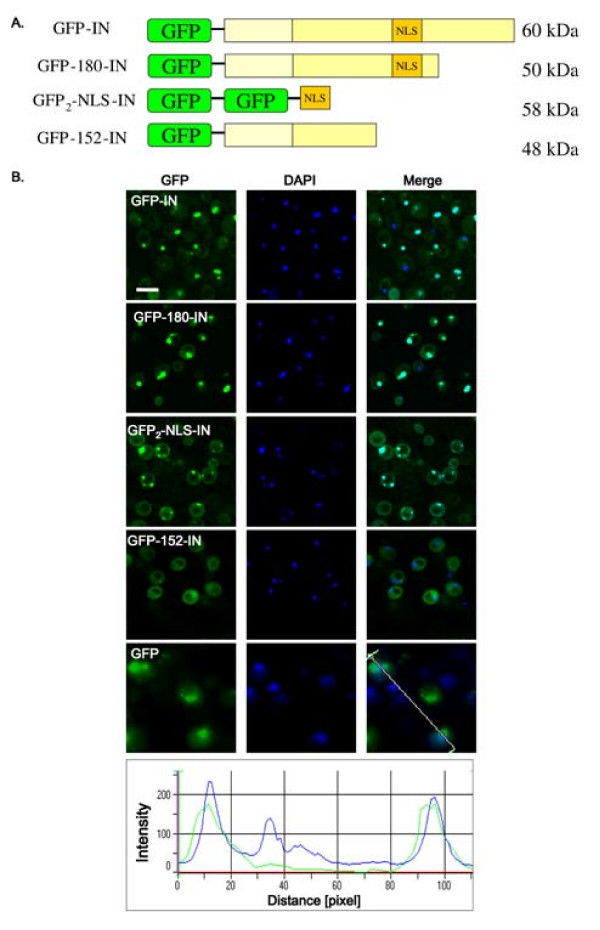
**Sub-cellular localization of the full-length and truncated IN fused to GFP in transformed yeast cells**. (A) Schematic presentation of the various expressed GFP-IN fusion proteins used in this experiment. (B) W303 yeast cells were transformed, using lithium acetate method, with expression vectors coding for the following: GFP-IN, GFP-180-IN, GFP_2_-NLS-IN, GFP-152-IN and GFP. Left panel, GFP fluorescence (green); middle panel, DAPI staining (blue); merged fluorescence is shown in the right panel. Bottom, a line profile through the overlay image showing that maximum GFP fluorescence and DAPI staining are co-localized (in the nucleus). Yeast cells were grown to exponential phase in selective minimal medium. After induction with galactose, cells were harvested and GFP fluorescence was observed under confocal microscope; all other conditions were as described in Methods. Bar 7 μm.

Due to the high ambiguity surrounding the nuclear import pathway of IN and its NLS domain, we studied its translocation into nuclei in a non-mammalian cell environment as well, namely in yeast cells. W303 cells were transformed with expression vectors encoding the full-length and truncated IN fused to the green fluorescence protein (GFP) (expressed proteins are schematized in Fig. 2A). As can be seen (Fig. 2B) in cells expressing GFP-IN, the fluorescence is packed into small intranuclear dots, as confirmed by DAPI staining of the cell's DNA, while both the cytosol and cell vacuoles appear relatively dark. The same was observed with the truncated GFP-180-IN, which includes the NLS-IN (Fig. 2B). Next, the ability of the NLS-IN sequence to promote nuclear import was studied. To create a molecule of high molecular weight, thereby avoiding passive nuclear import [2], the NLS-IN coding sequence was fused to the coding region of a double-GFP (GFP)_2 _(Fig. 2B). Similar to GFP-IN and GFP-180-IN, the expressed GFP_2_-NLS-IN fusion protein also accumulated within the yeast cell nuclei (Fig. 2B). In contrast, no nuclear import was observed in yeast cells transformed with an expression vector encoding the truncated GFP-152-IN, which lacks the putative NLS-IN (Fig. 2B). IN-mediated nuclear import can be inferred from the results showing that in yeast cells transformed with vectors expressing GFP molecules alone, the fluorescence distributed within the intracellular space (Fig. 2B). Yeast nuclei in all described experiments were identified by DAPI staining: GFP fluorescence appeared mostly in the nuclei (Fig. 2B).

**Figure 3:**
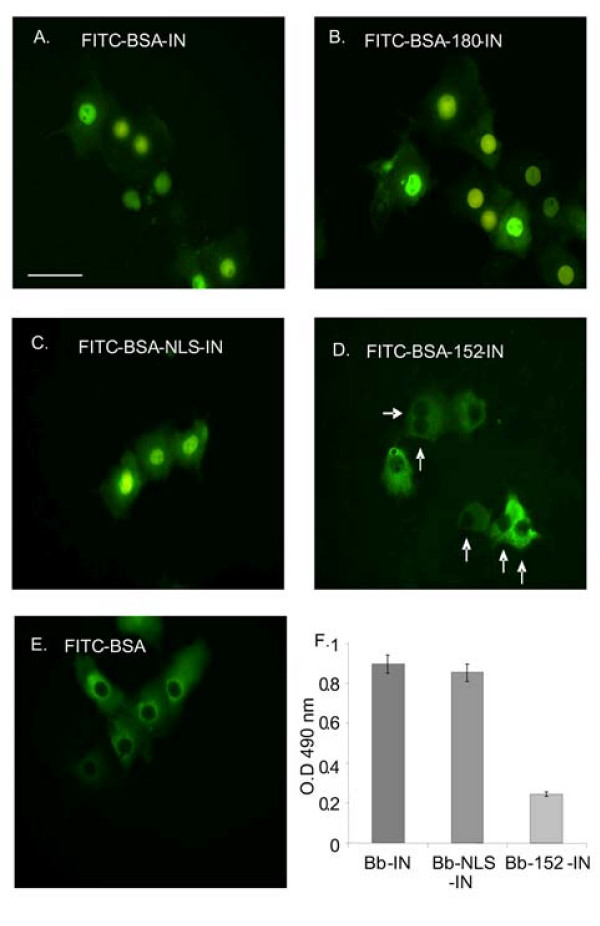
**Nuclear import mediated by recombinant HIV-1 IN protein: studies with microinjected and permeabilized mammalian cells**. Solutions containing the following conjugates: (A) FITC-BSA-IN, (B) FITC-BSA-180-IN, (C) FITC-BSA-NLS-IN, (D) FITC-BSA-152-IN and (E) FITC-BSA*, were microinjected into the cytoplasm of cultured COS-7 cells. All other experimental conditions were as described in Methods. (F) Nuclear import was quantitatively estimated by an ELISA-based assay system. Digitonin-permeabilized Colo-205 cells were incubated for 1 h with Bb-IN, Bb-NLS-IN or Bb-152-IN (4 μg) in a final volume of 40 μL of transport buffer containing ATP regeneration system. The nuclear import experiments were repeated at least three times; data given in the figure represent results obtained from a single experiment. Error bars represent standard deviation which is about +/-5%. Bar 10 μm.

Following the results in yeast cells, the karyophilic properties of the recombinant full-length IN and those of the truncated IN proteins were compared in microinjected cultured COS-7 cells. Microinjection of FITC-BSA-IN into COS-7 cells resulted in its translocation into the mammalian cells' nuclei (Fig. 3A). The same results were obtained following microinjection of FITC-BSA-180-IN or FITC-BSA-NLS-IN (Fig. 3B and 3C, respectively). On the other hand, very little, if any, nuclear import was observed when FITC-BSA-152-IN conjugates (truncated IN lacking the putative NLS-IN sequence) were microinjected into the COS-7 cells (see empty nuclei, arrows in Fig. 3D). Moreover, no nuclear import was observed when only FITC-BSA molecules were microinjected (Fig. 3E). It should be mentioned that the various recombinant IN proteins were attached to BSA molecules in order to increase their solubility as well as their molecular size, thus avoiding passive diffusion via the NPC.

Essentially similar results were obtained when the degree of nuclear import was quantitatively estimated using an ELISA-based system and biotinylated BSA (Bb) conjugates (Fig. 3F). A relatively high degree of nuclear import was observed with both Bb-NLS-IN and Bb-IN, whereas almost no import was observed with Bb-152-IN (Fig. 3E), again emphasizing the role of NLS-IN in mediating nuclear import of IN.

### NLS-IN mediates binding to importin α in yeast cells and *in vitro*

**Figure 4:**
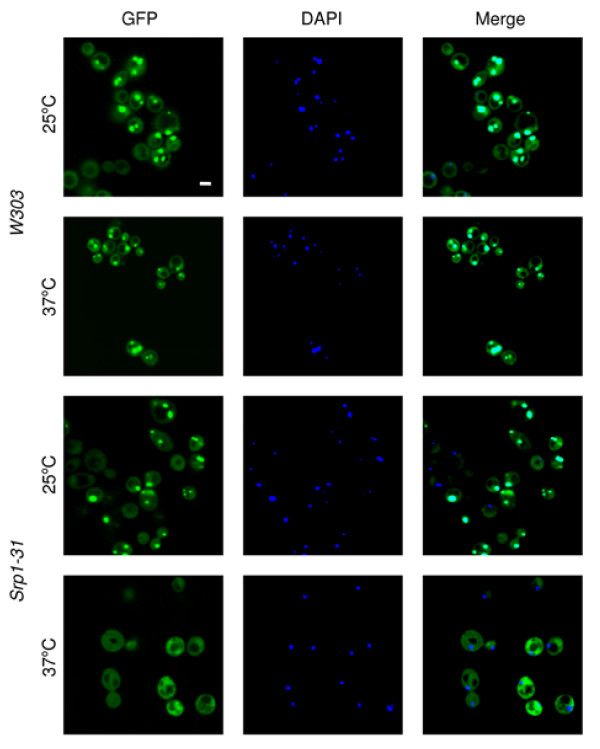
**Nuclear import of HIV-1 IN is importin α-dependent**. W303 and in *srp1-31 *yeast cells were transformed with plasmid bearing the full length of the IN fused to GFP (pYES_2_yEGFP-IN for the construction of the plasmid see Methods). Following transformation using the lithium acetate method, the fusion protein GFP-IN was expressed in the yeast cells, as described in Methods. GFP fluorescence (green) and DAPI (blue) were observed under confocal microscope following growth of W303 yeast cells at 25°C or at 37°C, or of *srp1-31 *yeast cells at 25°C or at the non-permissive temperature, 37°C. Bar 5 μm.

Our results showing inhibition of IN nuclear import by the SV40-NLS peptide indicated the involvement of importin α in the translocation process. To verify this, we used the yeast *srp1-31 *temperature-sensitive mutant [31] in which importin α is inactivated following growth at the non-permissive temperature of 37°C. GFP-IN appeared as small fluorescent dots when expressed in the wild-type W303 or in the *srp1-31 *mutant yeast cells grown at 25°C (Fig. 4). These results clearly demonstrate accumulation within the cells nuclei, a localization which was verified by DAPI staining. Neither the cytosol nor the cell vacuoles were strongly fluorescent. The same fluorescently stained dots were observed after 4 h growth of the W303 cells at 37°C (Fig. 4), again indicating accumulation of GFP-IN within the nuclei under these conditions. On the other hand, the *srp1-31 *mutant yeast cells lost their nuclear import ability at the non-permissive temperature (37°C): most of the GFP-IN was distributed within the yeast cell's cytoplasm (Fig. 4). However, nuclear localization was restored in these mutant cells following re-incubation at the permissive temperature of 25°C (not shown). To confirm that at the non-permissive temperature only importin α-dependent nuclear import is blocked, we repeated previous experiments in which Pik1 protein [36] had been shown to be imported into nuclei of *srp1-31 *cells at 37°C (not shown and see [36]), and in which it was established that nuclear import of this protein is importin α-independent [36]. Thus, the blockage is specific to the importin α pathway in *srp1-31 *cells at the non-permissive temperature.

**Figure 5:**
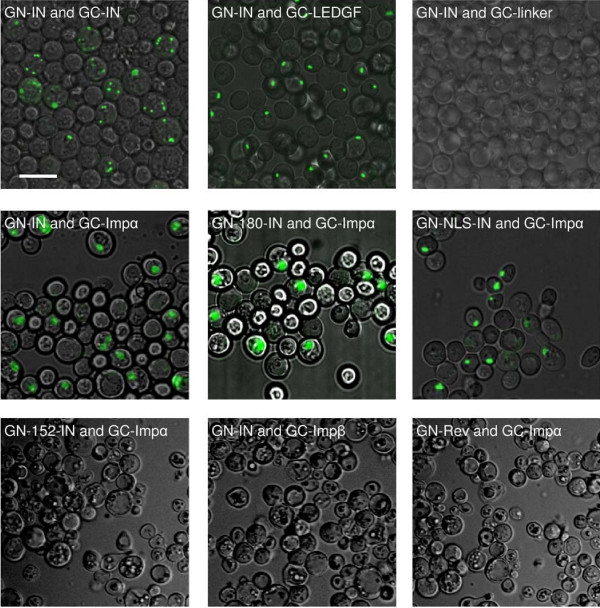
**IN interaction as observed by the BiFC assay system**. EGY48 yeast cells were transformed using the lithium acetate method with plasmids encoding the following combinations: GN-IN and GC-IN, GN-IN and GC-LEDGF, GN-IN and GC-linker (control), GN-IN and GC-Impα (importin α), GN-180-IN and GC-Impα, GN-NLS-IN and GC-Impα, GN-152-IN and GC-Impα, GN-IN and GC-Impβ (importin β), GN-Rev (HIV-1) and GC-Impα. Restoration of GFP fluorescence was observed by confocal microscopy. All other experimental conditions were as described in Methods. Bar 10 μm.

A specific IN-importin α interaction *in vivo *can be inferred also from the results obtained using the BiFC assay system in yeast cells ([32] and Fig. 5). As a control system, to confirm that restoration of fluorescence following the use of labeled IN is due to specific protein-protein interactions, the BiFC assay system was first employed to study the dimerization of the IN molecules themselves [37]. Indeed, intracellular fluorescence was seen in yeast cells which expressed both the GN-IN and GC-IN constructs (Fig. 5). No such fluorescence appeared in yeast cells expressing the combination of GN-IN and GC-linker (Fig. 5), or the combination of GN-linker and GC-IN (not shown), strengthening the view that the appearance of fluorescent dots resulted from specific IN-IN interaction. Next, we examined the interaction between the transcriptional co-activator LEDGF/p75 and IN (Fig. 5). LEDGF/p75 is the dominant cellular binding partner of HIV-1 IN in human cells [38]. Yeast cells were thus transformed with the combination of mammalian importin α (impα) and vectors expressing the various IN polypeptides. Fluorescence, indicating a direct interaction between GN-IN and GC-Impα, appeared in the yeast cell nuclei (Fig. 5). Nuclear fluorescence was also detected following transformation with either GN-180-IN or GN-NLS-IN and GC-Impα (Fig. 5). On the other hand, no fluorescence was detected in yeast cells transformed with the combination of GN-152-IN and GC-Impα (Fig. 5). Moreover, almost no complementation occurred in yeast cells transformed with the combination of GN-IN and GC-Impβ (importin β) (Fig. 5), again indicating that the appearance of fluorescence resulted from specific interaction of the IN with importin α. The same could be inferred from the negative results obtained following transformation of yeast cells with GN-Rev and GC-Impα: these yeast cells remained completely dark, with no fluorescent signal (Fig. 5). It has been well established that nuclear import of HIV-1 Rev is mediated by importin β and not α [39].

**Figure 6:**
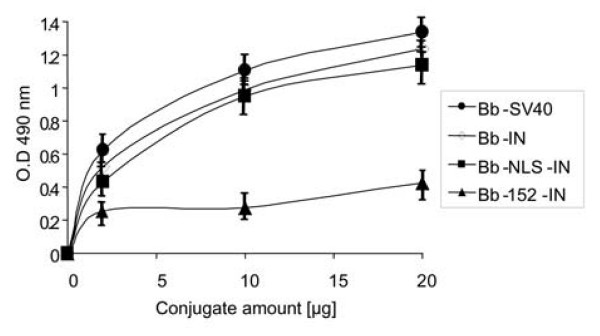
**Binding of IN to importin α as estimated by an ELISA-based system**. Importin α-coated ELISA plates were incubated with increasing amounts of the following biotinylated conjugates: SV40 (black circles), IN (white diamond), NLS-IN (black squares) and 152-IN (black triangles). The degree of binding was estimated as described in Methods. Error bars represent standard deviation which is about +/-5%.

Similar results were obtained when interactions were tested by the ELISA-based system with various IN conjugates and the receptor importin α. Our quantitative estimation revealed lower binding abilities by importin α with the Bb-152-IN conjugates as compared to the binding ability of Bb-IN, of Bb-NLS-IN and Bb-SV40-NLS conjugates (Fig. 6). These results again indicate that amino acids 161-173 are required for interaction with the importin α receptor.

### NLS-IN inhibits IN and cDNA nuclear import as well as HIV-1 replication in cultured cells

In the light of the results showing the requirement of NLS-IN for nuclear import of IN in mammalian as well as in yeast cells, it became of relevance to study its effect in virally infected cells.

**Figure 7:**
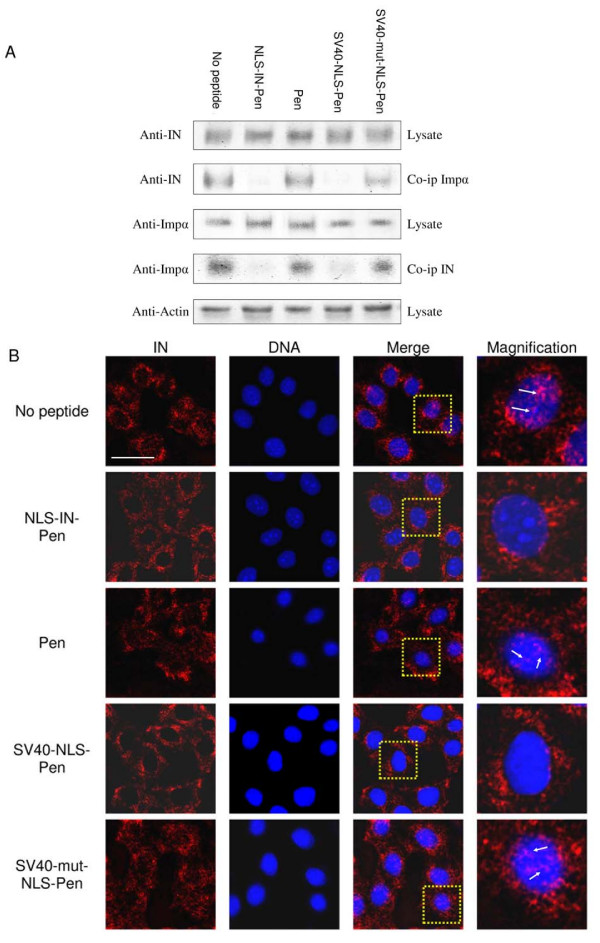
**NLS-IN-Pen inhibits IN nuclear import by the dissociation of IN-importin α interaction in HIV-infected cells**. (A) H9 lymphocytes were infected by wild-type HIV-1, and after infection half of the cells' lysate volume was subjected to SDS-PAGE, then immunoblotted with either by anti-IN, anti-importin α (anti-Impα) antibody or an anti-actin antibody. The complementary HRP-conjugated antibodies were used as the second antibody. The remaining lysate or isolated fractions were co-IP with either the anti-Impα or anti-IN antibodies and were immunoblotted with these antibodies, and the complementary HRP-conjugated antibodies as second antibodies. When peptides were used, cells were incubated with 150 μM of the indicated peptide. All others experimental details were as described in Methods. (B) HeLaP4 cells were infected and immunostained as described in Methods. IN (red); DAPI (blue); the area marked in the merge picture was magnified for a better view of IN localization within the infected cell. Bar 10 μm.

Co-immunoprecipitation (co-IP) experiments using a lysate obtained from HIV-1-infected cells revealed an interaction between the virus IN protein and the cellular importin α (Fig. 7A). Interestingly, when the virus-infected cells were treated with either the NLS-IN-Pen or the SV40-NLS-Pen peptides, no such interaction between the two proteins could be detected (Fig. 7A). Specificity of the peptide effect can be inferred from the results showing that neither the SV40-mut-NLS-Pen (or a scrambled NLS-IN [23], not shown) nor the Pen peptide itself promoted dissociation of the IN-importin α complex (Fig. 7A).

Inhibition of nuclear import of the viral IN in HIV-1 infected cells as well is evident from the immunofluorescence microscopic study shown in Fig. 7B. From the immunostaining results, it appears that in infected cells, the IN is localized both within the cytosol and within the nuclei (Fig. 7B (no peptide)). However, no intranuclear fluorescence was observed in cells treated with the NLS-IN-Pen or the SV40-NLS-Pen peptides, indicating the inhibition of nuclear import (Fig. 7B). In contrast, some intranuclear fluorescently labeled IN could be observed when the infected cells were incubated in the presence of the SV40-mut-NLS-Pen or the Pen peptide itself (Fig. 7B). This is also evident from the fact that more cytosolic IN was present in such peptide-treated cells than in those incubated in the absence of any peptide or with the non-active peptides (Fig. 7B).

**Figure 8:**
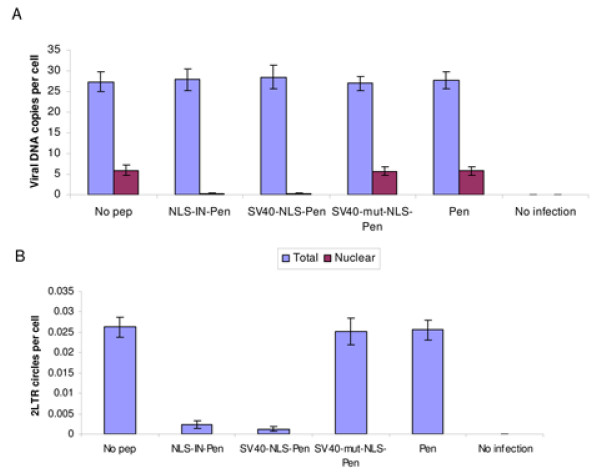
**NLS-IN-Pen inhibits nuclear import of viral DNA**. H9 lymphocytes were infected by wild-type HIV-1 at a MOI of 1; and (A) following infection, the nuclei fraction was isolated from half of the cells, and the amount of viral DNA was estimated using real time PCR method. (B) The amount of 2LTR circles was estimated using real time PCR method. All other experimental details are as described in Methods. Error bars represent standard deviation which is about +/-5%

Similar to their effect on IN nuclear import, both NLS-IN-Pen and SV40-NLS-Pen blocked nuclear import of the viral cDNA (Fig. 8A), as is particularly evident from the absence of 2LTR circles (Fig. 8B) in cells infected with wild-type HIV-1.

**Figure 9:**
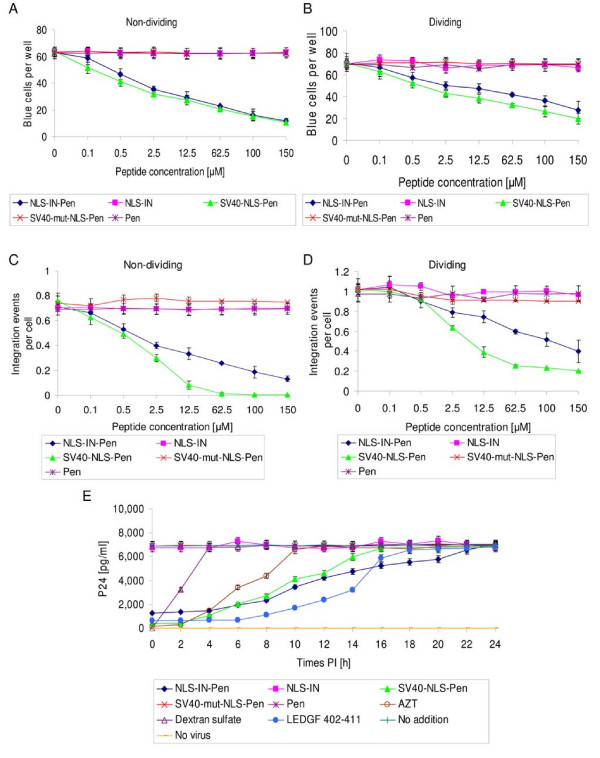
**NLS-IN-Pen peptide inhibits HIV-1**. (A) Cell-cycle arrested TZM-b1 cells (non-dividing cells) were incubated with the designated peptides at the indicated concentrations and after HIV-1 infection were tested for β-galactosidase activity. (B) Experimental conditions were as in (A), but with dividing TZM-b1 cells. The number of integration events per cell was determined in cell-cycle arrested (non-dividing) cells (C) or dividing cells (D) following incubation with the designated peptides at different concentrations. Cells were infected with HIV-1 at a MOI of 1 as described in Methods. (E) Inhibition of HIV-1 replication by NLS-IN-Pen as well as SV40-NLS-Pen is dependent on its time of addition. Sup-T1 cells were infected with HIV-1 at a MOI of 2, and the indicated elements were added at different time points after infection (0, 2, 4,..., 24 h). Viral p24 production was determined 48 h PI. Error bars represent standard deviation which is about +/-5%. All other experimental conditions are as described in Methods.

Inhibition of IN nuclear import is expected to result in the inhibition of virus replication, especially in cell-cycle arrested cells. Using TZM-bl cells [40] treated with aphidicolin to obtain cell-cycle arrested cells as an experimental system, a reduction in HIV-1 infection--as is reflected by the inhibition of reporter gene expression--was observed in the presence of NLS-IN-Pen or SV40-NLS-Pen (Fig. 9A). As expected, the inhibition was less pronounced when dividing cells were treated with the NLS-bearing peptides (Fig. 9B). The specific effect of NLS-IN and the requirement for cell permeability can be inferred from the results showing that no inhibition of HIV-1 infection was promoted by the Pen peptide alone or by the impermeable NLS-IN peptides. The results in Fig. 9C and 9D clearly demonstrate that the NLS-IN-Pen peptide due to its inhibitory effect on IN nuclear import inhibited the process of viral cDNA integration, reaching a higher degree of inhibition in non-dividing (cell-cycle arrested) than in dividing cells. Detailed kinetics studies (Fig. 9E) further support the view that the step which is blocked by the two NLS-Pen peptides (IN-NLS and SV40-NLS) is the nuclear import process. Evidently, nuclear import of the IN-DNA complex is required for the integration process to proceed. In addition, the time-dependent inhibitory pattern of the NLS-IN-Pen (Fig. 9E) is almost exactly half the way between that observed following the addition of AZT and that of the LEDGF 402-411 peptide, which has been demonstrated to directly block HIV-1 IN and thus the integration process [41]. Inhibition was not observed following the addition of the non-permeable NLS-IN peptide, a peptide bearing a SV40-mut-NLS-Pen or the Pen peptide, again indicating the specific effect of the NLS sequence (Fig. 9).

## Discussion

The question of how retroviruses and particularly HIV-1 cross the nuclear envelope in cell-cycle arrested cells is of specific scientific interest. After long and extensive research, it appears that no clear mechanism has yet emerged and the possibility that several pathways simultaneously mediate nuclear import of the viral PIC cannot be excluded. In the present work-following our previous experiments using *in vitro *systems [23]-we focused on the nuclear import of IN protein using yeast and mammalian cells, as well as on the contribution of its putative NLS-IN [23] on the HIV-1 replication process.

An interaction between HIV-1 IN and importin α was first demonstrated by Gallay *et al*. [8]. Similarly an interaction between the IN and members of the importin family and that its nuclear transport appears to be dependent on the importin α/β heterodimer have also demonstrated by Hearps and Jans [25]. A functional NLS sequence was identified between amino acids 161 and 173 of the IN protein whose mutation disrupted IN translocation into nuclei [12,42]. However, later studies indicated that this sequence may be required for promoting viral DNA integration and not necessarily nuclear import [43]. However, our previous work clearly demonstrated that a peptide bearing IN 161-173 residues can mediate import of a conjugated protein into the nuclei of permeabilized cells, confirming the view that it can function as a NLS [23].

In the present work, we have further studied the involvement of IN amino acids 161-173 in promoting its nuclear import. Nuclear accumulation was observed in yeast transformed with an expression vector bearing only the NLS-IN sequence, which in order to avoid diffusion into the nuclei, was fused to two molecules of GFP, resulting in a molecule of about 58 kDa. It is assumed that molecules of up to about 30 to 40 kDa can passively diffuse via the NPCs into cell nuclei [2]. Therefore, the nuclear import observed here with the various GFP-IN conjugates, the molecular weights of which varied between 48 and 60 kDa, should be ascribed to a receptor-mediated, active process. Nuclear import was practically not observed when yeast cells were transformed with the 152-IN truncated protein, which lacks the putative NLS sequence. Results obtained in permeabilized or microinjected cells, as well as in IN-transfected intact mammalian cells, further supported these results. The failure of 152-IN to penetrate the cell nuclei suggests that if an additional NLS sequence, besides the one located between 161 and 173, were present, it must be located closer to the C terminus of the IN protein, a possibility that has been suggested previously [25,44]. However, the fact that the truncated 180-IN protein was translocated into the cell nuclei indicates that the identified NLS-IN is sufficient to provide the IN with karyophilic properties. Our results demonstrating inhibition of IN nuclear import in transfected cells by a peptide carrying the NLS-IN sequence further emphasize the view that this sequence gives IN its karyophilic properties.

SRP1 is the only known importin α protein in budding yeast, and previous studies have demonstrated that it is essential for proper maintenance of nucleocytoplasmic trafficking [45]. Indeed, a temperature-sensitive mutant in SRP1 has been isolated (*srp1-31*) and shown to be defective in nuclear import at the non-permissive temperature [31]. Thus, yeast, including SRP1-mutated strains, has been instrumental in studying various aspects of the nuclear import machinery [36,46] and in characterizing karyophilic properties. The availability of the temperature-sensitive *srp1-31 *mutant offers an advantage for studying nuclear transport of the IN protein in yeast cells. The involvement of importin α in the IN nuclear import pathway can be inferred from our experiments showing nuclear import at the permissive, but not at the non-permissive temperature where importin α is inactivated [31].

A direct and specific interaction between IN or the NLS-IN sequence and the mammalian importin α *in vivo*, within the intracellular environment, was demonstrated using the BiFC assay. The view that restoration of GFP fluorescence using the BiFC assay in yeast cells results from a specific protein-protein interaction has already been established [47,48]. Indeed, our results clearly demonstrated the well-known IN-IN and IN-LEDGF/p75 interactions in yeast cells. Next we showed restoration of fluorescence in yeast cells expressing the combination of importin α and the full-length IN or the truncated 180-IN. The combination of importin α and the truncated 152-IN protein did not result in the appearance of fluorescence, strongly indicate that the NLS-IN sequence is located between amino acids 152 and 180, and is necessary to mediate the interaction with the nuclear receptor. However, our results do not exclude the possibility that additional NLS sequences are located between amino acid 180 and the C terminus, as suggested previously [25,44]. Attempts to study the interaction of fragments bearing these regions failed due to non-specific restoration of fluorescence (not shown). A similar pattern also characterized the *in vitro *interaction of the various INs. Our co-IP experiments confirmed the interaction between IN and importin α in virally infected cells. Furthermore, the view that such an interaction is mediated by the putative NLS-IN was supported by the results showing disruption of this interaction by the cell permeable NLS-IN or SV40-NLS peptides.

The NLS-IN-Pen peptide almost totally blocked viral infection of TZM-bl cells, caused high inhibition of the cDNA integration process, and affected p24 production when added up to 18 h post-infection (PI). Inhibitory effects were much higher in cell-cycle arrested than in dividing cells, clearly supporting the notion that the NLS-IN-Pen bears a sequence which is involved in mediating nuclear import of the IN. Inhibition of IN as well as of viral cDNA nuclear import by the NLS-IN-Pen peptide was demonstrated directly in the present work using immunofluorescence staining of IN and quantitative estimation of nuclear viral DNA.

The amino acid residues of NLS-IN described in the present work have been implicated in additional viral-related functions, such as specific binding of IN to the LEDGF/p75 protein and to the viral LTR region [38,49,50]. Due to its multifunctional activity, this region may be useful as a target for the development of inhibitors. As mentioned above our present as well as previous results describing the involvement of importin α in mediating nuclear import of IN do not exclude the possibility of an additional nuclear import pathway for IN in which TNPO3 is involved.

## Methods

### Mammalian, bacterial and yeast cells

Monolayer adherent HeLaP4 and HeLa TZM-bl cells (obtained through the NIH AIDS Research and Reference Reagent Program) expressing the β-galactosidase gene under regulation of the transactivation response element [51] were grown in Dulbecco's modified Eagle's medium. COS-7 and Colo-205 mammalian cells and the T-lymphocyte cell lines H9 and Sup-T1 were grown in RPMI 1640. All media were supplemented with 10% (v/v) fetal calf serum, 0.3 g/l L-glutamine, 100 units/ml penicillin, and 100 units/ml streptomycin (Biological Industries, Beit Haemek, Israel). Cells were incubated at 37°C in a 5% CO_2 _atmosphere and re-cultured every 4 days. HeLaP4/IN-expressing cells were generated by stable transfection into HeLaP4 cells [52] of pcDNA3.1 plasmid bearing the full wt IN gene. Selection was carried out for four weeks with 400 μg/ml Hygromycin B. *Escherichia coli *strain DH5α served as the host for general plasmid construction and maintenance, and *E. coli *strain BL21 (DE3) was used for protein overexpression. The yeast strains were congenic to *Saccharomyces cerevisiae *W303. The W303 (*MAT a, leu2-3, 112 trp1-1 ura3-1 ade2-1 his3-11,15*) and *srp1-31 *(*MAT a, leu2-3, 112 trp1-1 ura3-1 ade2-1 his3-11,15, srp1-31*) strains were kind gifts from G. Fink (Whitehead Institute, Massachusetts Institute of Technology, USA). The EGY48 strain (*MAT a, his3-11,15, trp1-1, ura3-52, leu2::LexA6op-LEU2*) was a kind gift from Y. Gafni (The Volcani Center, Israel). Yeast strains were grown in yeast peptone dextrose-rich medium (2% peptone, 2% glucose, 1% yeast extract, w/v). Transformation was performed by the lithium acetate method [53,54] and then the yeast cells were grown in standard yeast nitrogen (YNB) minimal media, prepared by adding the appropriate amino acids to 0.67% (w/v) YNB without amino acids (Difco) and supplemented with 2% glucose or 2% galactose (w/v) as carbon sources.

### Viruses

Wild-type HIV-1 was generated by transfection of HEK293T cells with pSVC21 plasmid containing the full-length HIV-1 HXB2 viral DNA. Wild-type viruses were harvested from HEK293T cells 48 and 72 h post-transfection. The viruses were stored at -75°C.

### Virus stock titration

Quantitative titration of HIV-1 was carried out using the MAGI assay, as described by Kimpton and Emerman [40]. Briefly, TZM-b1 cells were grown in 96-well plates at 1 × 10^4 ^cells per well. the cells were infected with 50 μl of serially diluted virus as described [40]. Two days post-infection (PI), cultured cells were fixed and β-galactosidase was estimated exactly as described previously [40]. Blue cells were counted under a light microscope at 200× magnification.

### Synthesis of peptides

Peptides were synthesized on Rink amide resin using a model 433A Applied Biosystems peptide synthesizer with FastMoc chemistry, exactly as described previously [55]. The Pen peptide had the following sequence: RQIKIWFQNRRMKWKK (Ant 43-58) [34].

### Plasmid construction

All of the plasmids used in this study were constructed using PCR cloning techniques with the high-fidelity enzyme Platinum *Pfx *DNA polymerase (Invitrogen). Clones were subjected to automated DNA sequencing.

#### 1. Site-directed mutagenesis to create a stop codon at position 152 or 180

The plasmid pT7-7-IN [56] was used as the template for IN mutagenesis. A QuikChange site-directed mutagenesis kit (Stratagene) was adapted to create an in-frame stop codon at the desired position within the IN sequence, according to the manufacturer's protocol. The mutagenic primers were designed to contain a stop codon after residues 152/180 of the IN giving pT7-7-152-IN/180-IN, respectively, as well as a new DraI site, to facilitate screening. The primers used were:

152-IN: 5'-CCCGCAGTCTCAGGGTGTTGTT**TAA**ACTATGAACAAAGAGCTC-3'

180-IN: 5'-CCGCGGTTCAGATGGCTGTT**TAA**ACCACAACAAGAAACG-3'

#### 2. Construction of plasmids for GFP in yeast cells

The yeast expression cloning plasmid pYES_2_yEGFP with the GFP codon optimized (a kind gift from T. Gilon, Alexander Silberman Institute, Israel) was linearized with the BsrGI restriction enzyme at the stop codon site of the yEGFP sequence [57], and then dephosphorylated and purified. The DNA products of IN, 152-IN, and 180-IN were obtained by PCR amplification from the pT7-7-152-IN/180-IN, respectively. The primers used were:

BsrGI, SalI-IN:

5'-CCGGCGTGTACAAAAGTCGACTAATGCACCACCATCACCAT-3'

IN-BamHI, BsrGI: 5'-GCCGGATGTACAGGATCCCCGGGCGCG-3'

These DNA products were then cloned into the BsrGI sites of the linearized pYES_2_yEGFP, resulting in the formation of pYES_2_yEGFP-IN (GFP-IN), pYES_2_yEGFP-152-IN (GFP-152-IN) and pYES_2_yEGFP-180-IN (GFP-180-IN). A GFP_2_-NLS-IN (amino acids 161 to 173 of IN) expression vector was prepared by PCR amplification from pT7-7-180-IN. The primers used were: 5'BsrGI: 5'-CCGCCATGTACAAAGAGCTCAAAAAAATCATCGGTCAG-3' 3 BsrGI: 5'-GCGGTATGTACACCAGCAGAGTAACCACCGATAC-3' The resultant DNA products were cloned into pBS-yEGFP, resulting in pBS-yEGFP-NLS-IN, which was digested by SalI and BamHI, and the resultant product was subcloned into pYES_2_yEGFP to give pYES_2_yEGFP_(2)_-NLS-IN (GFP_2_-NLS-IN).

#### 3. Construction of expression vectors for the BiFC assay

The yeast multicopy shuttle vectors pRS423 (with *HIS3 *as the selective marker) and pRS426 (with *URA3 *as the selective marker), both with the ADH1 promoter, were used as the cloning plasmids (a kind gift from D. Engelberg, Alexander Silberman Institute, Israel). The DNA coding region of the two GFP fragments [58], namely the N terminus (GN) and C terminus (GC), were cloned into pRS423 and pRS426 [59] to give pRS423-GN and pRS426-GC, respectively. A linker consisting of (GGS)_5 _was used to separate the inserted genes and the GFP fragments. The coding sequences of IN and HIV-1 Rev [60] were amplified by PCR, digested by XmaI and NotI and inserted in-frame into the corresponding sites of pRS423-GN at the C-terminal fragments of the GN, resulting in pRS423-GN-IN (GN-IN) and pRS423-GN-Rev (GN-Rev), respectively. The PCR products of 152-IN and 180-IN were ligated into the SalI and SacII sites of pRS423-GN to give pRS423-GN-152/180 (GN-152-IN/GN-180-IN). The plasmid pRS423-GN-NLS-IN (GN-NLS-IN) was obtained by PCR amplification of the NLS-IN sequence, digested by SacI and EcoRI and ligated at the corresponding sites of pRS423-GN. The PCR products of IN, importin α (hSRP1-α), importin β and LEDGF were cloned into the corresponding XmaI and NotI sites of pRS426-GC, to construct the following plasmids, respectively: pRS426-GC-IN (GC-IN), pRS426-GC-mammalian importin α (GC-Impα), pRS426-GC-importin β (GC-Impβ) and pRS426-GC-LEDGF (GC-LEDGF, PCR amplification from pET28-LEDGF a kind gift from C.A. Casiano, Loma Linda University, USA). The sequences of all primers used in this work can be obtained directly from the authors.

### Nuclear import of IN molecules in transformed yeast cells

Expression vectors bearing the GFP, GFP-IN, GFP-180-IN, GFP-152-IN, GFP_2_-NLS-IN and GFP-Pik1 coding regions under the galactose promoter (a generous gift from Dr. Thorner, UC Berkeley [36]) were introduced into the yeast strain W303. Expression was induced with 2% galactose for 4 h at 25°C. Following removal of the medium, the appearance of intracellular fluorescence was examined by confocal microscopy using an MRC 1024 confocal imaging system (Bio-Rad). Similarly, W303 and the *srp1-31 *yeast mutant were transformed by pYES2yEGFP-IN (GFP-IN). The strain *srp1-31 *contains a temperature-sensitive mutation in the SRP1 protein (importin α) [31,61]. The transformed yeast cells were grown for 24 h at 25°C to reach the logarithmic phase, then divided into two cultures: one remained at 25°C (permissive temperature) and the other was transferred to 37°C (non-permissive temperature at which more than 95% of the SRP1 is inactivated [61]). After 4 h of growth, yeast cells were washed; GFP-IN expression was induced by the addition of galactose and the yeast cells were incubated for an additional 4 h at either 25 or 37°C. At the end of the growth period, yeast cells were harvested and then observed by confocal microscope. Yeast nuclei were identified by DNA staining in fixed yeast cells (4% v/v paraformaldehyde and 3.4% w/v sucrose) http://www.ciwemb.edu/labs/koshland/Protocols/MICROSCOPY/gfpfix.html with 4',6-diamidino-2-phenylindole (DAPI). To confirm expression of all proteins, western blot with anti-GFP antibody was performed. Each experiment was repeated at least three times.

### Analysis of protein-protein interaction by the BiFC assay

In this approach, a molecule of GFP is separated into two portions: the N-terminal part (GN) ending at amino acid residue 154 and the C-terminal part (GC) beginning with the methionine residue preceding residue 155 of GFP [62]. Neither of these two halves of GFP fluoresce when expressed alone; however, the fluorescence is restored when GN and GC are brought together as fusions with interacting proteins [32]. The two halves of the GFP were cloned separately with each of the indicated proteins as described above and the different plasmids were transformed into the yeast strain EGY48. After 48 h at 30°C, the plates were transferred to 23°C for 3 days and then yeast cells were visualized by confocal microscopy. Nuclei were stained with DAPI as described above. Each experiment was repeated at least three times.

### Recombinant proteins

Expression and purification of the recombinant mammalian importin α (hSRP1-α) and HIV-1 IN proteins were performed essentially as described previously [23]. The vectors, as well as the methods used to obtain purified recombinant truncated IN bearing amino acids 1-180 (180-IN) or amino acids 1-152 (152-IN) were the same as described for the full-length IN [23].

### Nuclear import transport substrates

Proteins and peptides used in this work as transport substrates were covalently attached to either fluorescein isothiocyanate-labeled bovine serum albumin (FITC-BSA) or biotinylated BSA (Bb) molecules (Sigma). Sulfosuccinimidyl-4-(N-maleimidomethyl) cyclohexane-1-carboxylate (sulfo-SMCC) was used as the cross-linker to give FITC-BSA-protein/peptide and Bb-protein/peptide conjugates, as described previously [23].

### Binding of transport substrates to importin α

Binding to importin α was estimated using an ELISA-based system essentially as described in [23,63]. Briefly, Maxisorp plates (Nunc) were coated overnight at 4°C with a solution containing recombinant importin α (5 μg) in carbonate buffer (NaHCO_3_/Na_2_CO_3 _buffer at pH 9.6). All subsequent steps were as described previously [23].

### Microinjection of fluorescently labeled transport substrates

FITC-BSA-IN, FITC-BSA-180-IN, FITC-BSA-152-IN, FITC-BSA-NLS-IN, as well as unlabeled FITC-BSA, were microinjected into the cytoplasm of cultured COS-7 cells as described previously [64]. The CompInject AIS2 automated microinjection system (Cell Biology Trading, Hamburg, Germany) was used following the method developed by Graessmann and Graessman [65]. Cultured mammalian cells were incubated at 37°C for 2 h after injection and observed by fluorescence microscopy.

### Nuclear import in permeabilized mammalian cells: quantitative estimation

Import of Bb-IN proteins or of Bb-NLS-IN conjugates into nuclei of digitonin-permeabilized Colo-205 cells was determined quantitatively by an ELISA-based system, exactly as described previously [23].

### Localization studies of transfected IN in cultured cells by immunostaining

HeLaP4/IN-expressing cells were grown on chamber slides (Nunc). After reaching 70-80% confluence, cells were arrested in the cell cycle by treatment with 5 μg/ml of aphidicolin, and then incubated with 150 μM of the indicated peptide for 6 h. Cells were fixed and immunostained essentially as described previously [66] with several modifications. Briefly, after fixation cells were blocked with 5% (w/v) BSA (IgG free) (Jackson) in PBS for 60 min. For detection of HIV-1 IN, cells were incubated with 1:100 rabbit α-IN (NIH AIDS Research & Reference Reagent Program, cat. no. 758) at room temperature for 60 min each. Cells were washed five times with PBS + 0.05% (v/v) Tween 20. Then the cells were incubated with a second antibody, Cy3-conjugated anti-rabbit antibody (Jackson) (1:200) at room temperature for 60 min followed by five washes with PBS + 0.05% Tween 20. For detection of DNA, cells were stained with DAPI according to the manufacturer's protocol. The slides were prepared with mounting media (Bio-Rad) and immunofluorescent cells were detected with a confocal microscope.

### Localization studies of IN in HIV-1-infected cultured cells by immunostaining

HeLaP4 cells were grown on chamber slides. Cells were arrested in the cell cycle by treatment with 5 μg/ml aphidicolin, and then incubated with 150 μM of the indicated peptide for 2 h. After incubation with the peptides, cells were infected with wild-type HIV-1 at a multiplicity of infection (MOI) of 25. Cells were fixed and stained as described above with the following modifications: fixation was performed at 10 h PI, the first antibody was used at a dilution of 1:50, the second antibody at a dilution of 1:100.

### Study of *in vivo *protein-protein interactions using co-IP

Cells were infected with a MOI of 15 of the indicated viruses, harvested at 10 h post-infection (PI), washed three times with PBS and lysed by the addition of PBS containing 1% (v/v) Triton X-100. Half of the lysate volume was subjected to SDS-PAGE, then immunoblotted with either antiserum raised against IN amino acids 276-288 (anti-IN) (NIH AIDS Research & Reference Reagent Program cat. no. 758), anti-importin α (anti-Impα) antibody (Santa Cruz), or an anti-actin (anti-Actin) antibody (Santa Cruz) The complementary HRP-conjugated antibodies (Jackson) were used as the second antibody.

The remaining lysate or isolated fractions were incubated for 1 h at 4°C with either the anti-Impα or anti-IN antibodies. Following 3 h incubation with protein G-agarose beads (Santa Cruz) at 4°C, the samples were washed three times with PBS containing 1% (v/v) Nonidet P-40. SDS buffer was added to the samples and after boiling and running on an SDS polyacrylamide gel, the membranes were immunoblotted with either anti-Impα or anti-IN antibodies, and the complementary HRP-conjugated antibodies (Jackson) as second antibodies.

When peptides were used, cells were incubated with 150 μM of the indicated peptide for 2 h prior to infection.

### HIV-1 titration by multinuclear activation of a galactosidase indicator (MAGI) assay

Quantitative titration of HIV-1 was carried out using the MAGI assay, as described previously [40]. Briefly, TZM-b1 cells were grown in 96-well plates at 10^4 ^cells/well and incubated for 12 h at 37°C. Peptides were then added and after an additional 2 h of incubation, the cells were infected with 50 μl of serially diluted HIV-1. To obtain cell-cycle arrested cells, 5 μg/ml of aphidicolin was added 2 h before the experiment. Cultured cells were fixed 2 days PI and β-galactosidase was estimated [47]. Blue cells were counted under a light microscope at 200× magnification.

### Quantitative analysis of integration

Real-time PCR experiments were performed to estimate integration as described previously [67].

### Time-of-addition assay

Sup-T1 cells were infected with wild-type HIV-1 at a MOI of 2, and the test compounds were added at different time points after infection (0, 2, 4,..., 24 h). Viral p24 production was determined at 48 h PI [67]. Dextran sulfate was tested at 20 μM, AZT at 2 μM, NLS-IN-Pen, NLS-IN and Pen at 62.5 μM, LEDGF 402-411 at 12.5 μM [41].

### Isolation of cytoplasm and nuclei from infected cells

The various fractions were obtained from virus-infected cells essentially as described previously [68,69] with several modifications. Briefly, cells were harvested and washed twice in buffer A (20 mM Hepes pH 7.3, 150 mM KCl, 5 mM MgCl_2_, 1 mM DTT and 0.1 mM PMSF). Cells were then suspended in 200 μl of buffer A with 0.025% (w/v) digitonin and incubated at room temperature for 10 min. Cells were centrifuged for 3 min at 1000 *g *at room temperature. The supernatant was then centrifuged at 8000 *g *and separated into supernatant (cytoplasm) and pellet (nuclei) and stored at -70°C.

### Quantitation of total and nuclear viral DNA

Total viral DNA was estimated using SYBR green real-time quantitative PCR at 10 h PI from the total or nuclear-isolated fractions of the infected cells. DNA was isolated by phenol chloroform method. Briefly, DNA samples (1 μg of DNA) were added to 95 μl containing 1× Hot-Rescue Real Time PCR Kit-SG (Diatheva s.r.l, Fano, Italy), and 100 nM of each PBS (primer-binding site) primer: F5 (5' primer, 5'-TAGCAGTGGCGCCCGA-3') and R5 (3' primer, 5-TCTCTCTCCTTCTAGCCTCCGC-3'). All amplification reactions were carried out using an ABI Prism 7700 Sequence Detection System (Applied Biosystems): One cycle at 95°C for 10 min, followed by 45 cycles of 15 s at 95°C and 35 s at 68°C. In each PCR run, three replicates were performed. All other details are exactly as described in Casabianca *et al*. [70].

### Quantitation of 2LTR circles

Quantification of 2LTR circles was estimated exactly as described in Butler *et al*. [71].

## Competing interests

The authors declare that they have no competing interests.

## Authors' contributions

ALevin designed and performed experiments (immunostaining, co-IP and HIV-1 tests), analyzed data and contributed to writing the paper; AAO designed and performed experiments, analyzed data and contributed to writing the paper; JR contributed to the study design of the BiFC assay; NMB provided technical support and contributed to the confocal images; AG performed the microinjection experiments; EW evaluated the manuscript; ALoyter designed the study, contributed to writing the paper and coordinated the study. All authors have read and approved the manuscript.

## References

[B1] Stewart M Molecular mechanism of the nuclear protein import cycle Nat Rev Mol Cell Biol 2007 8 195 208 17287812 10.1038/nrm2114

[B2] Gorlich D Kutay U Transport between the cell nucleus and the cytoplasm Annu Rev Cell Dev Biol 1999 15 607 660 10611974 10.1146/annurev.cellbio.15.1.607

[B3] Feldherr C Akin D Littlewood T Stewart M The molecular mechanism of translocation through the nuclear pore complex is highly conserved J Cell Sci 2002 115 2997 3005 12082159 10.1242/jcs.115.14.2997

[B4] Lewis P Hensel M Emerman M Human immunodeficiency virus infection of cells arrested in the cell cycle EMBO J 1992 11 3053 3058 1322294 10.1002/j.1460-2075.1992.tb05376.x PMC556788

[B5] Katz RA Greger JG Boimel P Skalka AM Human immunodeficiency virus type 1 DNA nuclear import and integration are mitosis independent in cycling cells J Virol 2003 77 13412 13417 14645598 10.1128/JVI.77.24.13412-13417.2003 PMC296078

[B6] Miller MD Farnet CM Bushman FD Human immunodeficiency virus type 1 preintegration complexes: studies of organization and composition J Virol 1997 71 5382 5390 9188609 10.1128/jvi.71.7.5382-5390.1997 PMC191777

[B7] Suzuki Y Craigie R The road to chromatin - nuclear entry of retroviruses Nat Rev Microbiol 2007 5 187 196 17304248 10.1038/nrmicro1579

[B8] Gallay P Hope T Chin D Trono D HIV-1 infection of nondividing cells through the recognition of integrase by the importin/karyopherin pathway Proc Natl Acad Sci USA 1997 94 9825 9830 9275210 10.1073/pnas.94.18.9825 PMC23276

[B9] Haffar OK Popov S Dubrovsky L Agostini I Tang H Pushkarsky T Nadler SG Bukrinsky M Two nuclear localization signals in the HIV-1 matrix protein regulate nuclear import of the HIV-1 pre-integration complex J Mol Biol 2000 299 359 368 10860744 10.1006/jmbi.2000.3768

[B10] Jenkins Y McEntee M Weis K Greene WC Characterization of HIV-1 vpr nuclear import: analysis of signals and pathways J Cell Biol 1998 143 875 885 9817747 10.1083/jcb.143.4.875 PMC2132945

[B11] Zennou V Petit C Guetard D Nerhbass U Montagnier L Charneau P HIV-1 genome nuclear import is mediated by a central DNA flap Cell 2000 101 173 185 10786833 10.1016/S0092-8674(00)80828-4

[B12] Llano M Vanegas M Fregoso O Saenz D Chung S Peretz M Poeschla EM LEDGF/p75 determines cellular trafficking of diverse lentiviral but not murine oncoretroviral integrase proteins and is a component of functional lentiviral preintegration complexes J Virol 2004 78 9524 9537 15308744 10.1128/JVI.78.17.9524-9537.2004 PMC506940

[B13] Maertens G Cherepanov P Pluymers W Busschots K De Clercq E Debyser Z Engelborghs Y LEDGF/p75 is essential for nuclear and chromosomal targeting of HIV-1 integrase in human cells J Biol Chem 2003 278 33528 33539 12796494 10.1074/jbc.M303594200

[B14] Yamashita M Perez O Hope TJ Emerman M Evidence for direct involvement of the capsid protein in HIV infection of nondividing cells PLoS Pathog 2007 3 1502 1510 17967060 10.1371/journal.ppat.0030156 PMC2042020

[B15] Sherman MP Greene WC Slipping through the door: HIV entry into the nucleus Microbes Infect 2002 4 67 73 11825777 10.1016/s1286-4579(01)01511-8

[B16] Woodward CL Wang Y Dixon WJ Htun H Chow SA Subcellular localization of feline immunodeficiency virus integrase and mapping of its karyophilic determinant J Virol 2003 77 4516 4527 12663758 10.1128/JVI.77.8.4516-4527.2003 PMC152119

[B17] Zheng R Jenkins TM Craigie R Zinc folds the N-terminal domain of HIV-1 integrase, promotes multimerization, and enhances catalytic activity Proc Natl Acad Sci USA 1996 93 13659 13664 8942990 10.1073/pnas.93.24.13659 PMC19383

[B18] Kulkosky J Jones KS Katz RA Mack JP Skalka AM Residues critical for retroviral integrative recombination in a region that is highly conserved among retroviral/retrotransposon integrases and bacterial insertion sequence transposases Mol Cell Biol 1992 12 2331 2338 1314954 10.1128/mcb.12.5.2331-2338.1992 PMC364405

[B19] Chiu TK Davies DR Structure and function of HIV-1 integrase Curr Top Med Chem 2004 4 965 977 15134551 10.2174/1568026043388547

[B20] Esposito D Craigie R HIV integrase structure and function Adv Virus Res 1999 52 319 333 10384240 10.1016/s0065-3527(08)60304-8

[B21] Chen Z Yan Y Munshi S Li Y Zugay-Murphy J Xu B Witmer M Felock P Wolfe A Sardana V Emini EA Hazuda D Kuo LC X-ray structure of simian immunodeficiency virus integrase containing the core and C-terminal domain (residues 50-293)--an initial glance of the viral DNA binding platform J Mol Biol 2000 296 521 533 10669606 10.1006/jmbi.1999.3451

[B22] Pluymers W Cherepanov P Schols D De Clercq E Debyser Z Nuclear localization of human immunodeficiency virus type 1 integrase expressed as a fusion protein with green fluorescent protein Virology 1999 258 327 332 10366569 10.1006/viro.1999.9727

[B23] Armon-Omer A Graessmann A Loyter A A synthetic peptide bearing the HIV-1 integrase 161-173 amino acid residues mediates active nuclear import and binding to importin alpha: characterization of a functional nuclear localization signal J Mol Biol 2004 336 1117 1128 15037073 10.1016/j.jmb.2003.11.057

[B24] Depienne C Mousnier A Leh H Le Rouzic E Dormont D Benichou S Dargemont C Characterization of the nuclear import pathway for HIV-1 integrase J Biol Chem 2001 276 18102 18107 11278458 10.1074/jbc.M009029200

[B25] Hearps AC Jans DA HIV-1 integrase is capable of targeting DNA to the nucleus via an importin alpha/beta-dependent mechanism Biochem J 2006 398 475 484 16716146 10.1042/BJ20060466 PMC1559465

[B26] Ao Z Huang G Yao H Xu Z Labine M Cochrane AW Yao X Interaction of human immunodeficiency virus type 1 integrase with cellular nuclear import receptor importin 7 and its impact on viral replication J Biol Chem 2007 282 13456 13467 17360709 10.1074/jbc.M610546200

[B27] Christ F Thys W De Rijck J Gijsbers R Albanese A Arosio D Emiliani S Rain JC Benarous R Cereseto A Debyser Z Transportin-SR2 imports HIV into the nucleus Curr Biol 2008 18 1192 1202 18722123 10.1016/j.cub.2008.07.079

[B28] Emiliani S Mousnier A Busschots K Maroun M Van Maele B Tempe D Vandekerckhove L Moisant F Ben-Slama L Witvrouw M Christ F Rain JC Dargemont C Debyser Z Benarous R Integrase mutants defective for interaction with LEDGF/p75 are impaired in chromosome tethering and HIV-1 replication J Biol Chem 2005 280 25517 25523 15855167 10.1074/jbc.M501378200

[B29] Zielske SP Stevenson M Importin 7 may be dispensable for human immunodeficiency virus type 1 and simian immunodeficiency virus infection of primary macrophages J Virol 2005 79 11541 11546 16103209 10.1128/JVI.79.17.11541-11546.2005 PMC1193637

[B30] Zaitseva L Cherepanov P Leyens L Wilson SJ Rasaiyaah J Fassati A HIV-1 exploits importin 7 to maximize nuclear import of its DNA genome Retrovirology 2009 6 11 19193229 10.1186/1742-4690-6-11 PMC2660290

[B31] Yano R Oakes M Yamaghishi M Dodd JA Nomura M Cloning and characterization of SRP1, a suppressor of temperature-sensitive RNA polymerase I mutations, in Saccharomyces cerevisiae Mol Cell Biol 1992 12 5640 5651 1448093 10.1128/mcb.12.12.5640-5651.1992 PMC360503

[B32] Kerppola TK Visualization of molecular interactions by fluorescence complementation Nat Rev Mol Cell Biol 2006 7 449 456 16625152 10.1038/nrm1929 PMC2512262

[B33] Bouyac-Bertoia M Dvorin JD Fouchier RA Jenkins Y Meyer BE Wu LI Emerman M Malim MH HIV-1 infection requires a functional integrase NLS Mol Cell 2001 7 1025 1035 11389849 10.1016/s1097-2765(01)00240-4

[B34] Pietersz GA Li W Apostolopoulos V A 16-mer peptide (RQIKIWFQNRRMKWKK) from antennapedia preferentially targets the Class I pathway Vaccine 2001 19 1397 1405 11163662 10.1016/s0264-410x(00)00373-x

[B35] Conti E Kuriyan J Crystallographic analysis of the specific yet versatile recognition of distinct nuclear localization signals by karyopherin alpha Structure Fold Des 2000 8 329 338 10745017 10.1016/s0969-2126(00)00107-6

[B36] Strahl T Hama H DeWald DB Thorner J Yeast phosphatidylinositol 4-kinase, Pik1, has essential roles at the Golgi and in the nucleus J Cell Biol 2005 171 967 979 16365163 10.1083/jcb.200504104 PMC1382337

[B37] Kalpana GV Goff SP Genetic analysis of homomeric interactions of human immunodeficiency virus type 1 integrase using the yeast two-hybrid system Proc Natl Acad Sci USA 1993 90 10593 10597 8248150 10.1073/pnas.90.22.10593 PMC47823

[B38] Cherepanov P Ambrosio AL Rahman S Ellenberger T Engelman A Structural basis for the recognition between HIV-1 integrase and transcriptional coactivator p75 Proc Natl Acad Sci USA 2005 102 17308 17313 16260736 10.1073/pnas.0506924102 PMC1297672

[B39] Truant R Cullen BR The arginine-rich domains present in human immunodeficiency virus type 1 Tat and Rev function as direct importin beta-dependent nuclear localization signals Mol Cell Biol 1999 19 1210 1217 9891055 10.1128/mcb.19.2.1210 PMC116050

[B40] Kimpton J Emerman M Detection of replication-competent and pseudotyped human immunodeficiency virus with a sensitive cell line on the basis of activation of an integrated beta-galactosidase gene J Virol 1992 66 2232 2239 1548759 10.1128/jvi.66.4.2232-2239.1992 PMC289016

[B41] Hayouka Z Rosenbluh J Levin A Loya S Lebendiker M Veprintsev D Kotler M Hizi A Loyter A Friedler A Inhibiting HIV-1 integrase by shifting its oligomerization equilibrium Proc Natl Acad Sci USA 2007 104 8316 8321 17488811 10.1073/pnas.0700781104 PMC1895947

[B42] Dvorin JD Bell P Maul GG Yamashita M Emerman M Malim MH Reassessment of the roles of integrase and the central DNA flap in human immunodeficiency virus type 1 nuclear import J Virol 2002 76 12087 12096 12414950 10.1128/JVI.76.23.12087-12096.2002 PMC136890

[B43] Limon A Devroe E Lu R Ghory HZ Silver PA Engelman A Nuclear localization of human immunodeficiency virus type 1 preintegration complexes (PICs): V165A and R166A are pleiotropic integrase mutants primarily defective for integration, not PIC nuclear import J Virol 2002 76 10598 10607 12368302 10.1128/JVI.76.21.10598-10607.2002 PMC136649

[B44] Ao Z Fowke KR Cohen EA Yao X Contribution of the C-terminal tri-lysine regions of human immunodeficiency virus type 1 integrase for efficient reverse transcription and viral DNA nuclear import Retrovirology 2005 2 62 16232319 10.1186/1742-4690-2-62 PMC1277849

[B45] Silver P Sadler I Osborne MA Yeast proteins that recognize nuclear localization sequences J Cell Biol 1989 109 983 989 2670959 10.1083/jcb.109.3.983 PMC2115749

[B46] Wendler P Lehmann A Janek K Baumgart S Enenkel C The bipartite nuclear localization sequence of Rpn2 is required for nuclear import of proteasomal base complexes via karyopherin alphabeta and proteasome functions J Biol Chem 2004 279 37751 37762 15210724 10.1074/jbc.M403551200

[B47] Rosenbluh J Hayouka Z Loya S Levin A Armon-Omer A Britan E Hizi A Kotler M Friedler A Loyter A Interaction between HIV-1 Rev and Integrase Proteins: A BASIS FOR THE DEVELOPMENT OF ANTI-HIV PEPTIDES J Biol Chem 2007 282 15743 15753 17403681 10.1074/jbc.M609864200

[B48] Cole KC McLaughlin HW Johnson DI Use of bimolecular fluorescence complementation to study in vivo interactions between Cdc42p and Rdi1p of Saccharomyces cerevisiae Eukaryot Cell 2007 6 378 387 17220465 10.1128/EC.00368-06 PMC1828923

[B49] Chen A Weber IT Harrison RW Leis J Identification of amino acids in HIV-1 and avian sarcoma virus integrase subsites required for specific recognition of the long terminal repeat Ends J Biol Chem 2006 281 4173 4182 16298997 10.1074/jbc.M510628200 PMC2656937

[B50] Xu Z Zheng Y Ao Z Clement M Mouland AJ Kalpana GV Belhumeur P Cohen EA Yao X Contribution of the C-terminal region within the catalytic core domain of HIV-1 integrase to yeast lethality, chromatin binding and viral replication Retrovirology 2008 5 102 19014595 10.1186/1742-4690-5-102 PMC2615443

[B51] Derdeyn CA Decker JM Sfakianos JN Wu X O'Brien WA Ratner L Kappes JC Shaw GM Hunter E Sensitivity of human immunodeficiency virus type 1 to the fusion inhibitor T-20 is modulated by coreceptor specificity defined by the V3 loop of gp120 J Virol 2000 74 8358 8367 10954535 10.1128/jvi.74.18.8358-8367.2000 PMC116346

[B52] Cullen BR Use of eukaryotic expression technology in the functional analysis of cloned genes Methods Enzymol 1987 152 684 704 3657593 10.1016/0076-6879(87)52074-2

[B53] Schiestl RH Gietz RD High efficiency transformation of intact yeast cells using single stranded nucleic acids as a carrier Curr Genet 1989 16 339 346 2692852 10.1007/BF00340712

[B54] Gietz D St Jean A Woods RA Schiestl RH Improved method for high efficiency transformation of intact yeast cells Nucleic Acids Res 1992 20 1425 1561104 10.1093/nar/20.6.1425 PMC312198

[B55] Armon-Omer A Levin A Hayouka Z Butz K Hoppe-Seyler F Loya S Hizi A Friedler A Loyter A Correlation between shiftide activity and HIV-1 integrase inhibition by a peptide selected from a combinatorial library J Mol Biol 2008 376 971 982 18201721 10.1016/j.jmb.2007.11.095

[B56] Chen JC Krucinski J Miercke LJ Finer-Moore JS Tang AH Leavitt AD Stroud RM Crystal structure of the HIV-1 integrase catalytic core and C-terminal domains: a model for viral DNA binding Proc Natl Acad Sci USA 2000 97 8233 8238 10890912 10.1073/pnas.150220297 PMC26930

[B57] Cormack BP Bertram G Egerton M Gow NA Falkow S Brown AJ Yeast-enhanced green fluorescent protein (yEGFP) a reporter of gene expression in Candida albicans Microbiology 1997 143 Pt 2 303 311 9043107 10.1099/00221287-143-2-303

[B58] Magliery TJ Wilson CG Pan W Mishler D Ghosh I Hamilton AD Regan L Detecting protein-protein interactions with a green fluorescent protein fragment reassembly trap: scope and mechanism J Am Chem Soc 2005 127 146 157 15631464 10.1021/ja046699g

[B59] Christianson TW Sikorski RS Dante M Shero JH Hieter P Multifunctional yeast high-copy-number shuttle vectors Gene 1992 110 119 122 1544568 10.1016/0378-1119(92)90454-w

[B60] Fineberg K Fineberg T Graessmann A Luedtke NW Tor Y Lixin R Jans DA Loyter A Inhibition of nuclear import mediated by the Rev-arginine rich motif by RNA molecules Biochemistry 2003 42 2625 2633 12614157 10.1021/bi0206199

[B61] Loeb JD Schlenstedt G Pellman D Kornitzer D Silver PA Fink GR The yeast nuclear import receptor is required for mitosis Proc Natl Acad Sci USA 1995 92 7647 7651 7644471 10.1073/pnas.92.17.7647 PMC41202

[B62] Hu CD Chinenov Y Kerppola TK Visualization of interactions among bZIP and Rel family proteins in living cells using bimolecular fluorescence complementation Mol Cell 2002 9 789 798 11983170 10.1016/s1097-2765(02)00496-3

[B63] Rosenbluh J Kapelnikov A Shalev DE Rusnati M Bugatti A Loyter A Positively charged peptides can interact with each other, as revealed by solid phase binding assays Anal Biochem 2006 352 157 168 16581010 10.1016/j.ab.2006.03.002

[B64] Hariton-Gazal E Feder R Mor A Graessmann A Brack-Werner R Jans D Gilon C Loyter A Targeting of nonkaryophilic cell-permeable peptides into the nuclei of intact cells by covalently attached nuclear localization signals Biochemistry 2002 41 9208 9214 12119035 10.1021/bi0201466

[B65] Graessmann M Graessmann A Microinjection of tissue culture cells Methods Enzymol 1983 101 482 492 6310338 10.1016/0076-6879(83)01033-2

[B66] Levin A Kutznetova L Kahana R Rubinstein-Guini M Stram Y Highly effective inhibition of Akabane virus replication by siRNA genes Virus Res 2006 120 121 127 16616390 10.1016/j.virusres.2006.02.009

[B67] Levin A Hayouka Z Helfer M Brack-Werner R Friedler A Loyter A Peptides derived from HIV-1 integrase that bind Rev stimulate viral genome integration PLoS ONE 2009 4 e4155 19127291 10.1371/journal.pone.0004155 PMC2607543

[B68] Zhang J Scadden DT Crumpacker CS Primitive hematopoietic cells resist HIV-1 infection via p21 J Clin Invest 2007 117 473 481 17273559 10.1172/JCI28971 PMC1783820

[B69] Levin A Rosenbluh J Hayouka Z Friedler A Loyter A Integration of HIV-1 DNA is regulated by interplay between viral Rev and cellular LEDGF/p75 proteins Mol Med 2009 in press 19855849 10.2119/molmed.2009.00133 PMC2765407

[B70] Casabianca A Gori C Orlandi C Forbici F Federico Perno C Magnani M Fast and sensitive quantitative detection of HIV DNA in whole blood leucocytes by SYBR green I real-time PCR assay Mol Cell Probes 2007 21 368 378 17629450 10.1016/j.mcp.2007.05.005

[B71] Butler SL Hansen MS Bushman FD A quantitative assay for HIV DNA integration in vivo Nat Med 2001 7 631 634 11329067 10.1038/87979

